# Deep Sequencing of Antiviral T-Cell Responses to HCMV and EBV in Humans Reveals a Stable Repertoire That Is Maintained for Many Years

**DOI:** 10.1371/journal.ppat.1002889

**Published:** 2012-09-27

**Authors:** P. L. Klarenbeek, E. B. M. Remmerswaal, I. J. M. ten Berge, M. E. Doorenspleet, B. D. C. van Schaik, R. E. E. Esveldt, S. D. Koch, A. ten Brinke, A. H. C. van Kampen, F. J. Bemelman, P. P. Tak, F. Baas, N. de Vries, R. A. W. van Lier

**Affiliations:** 1 Department of Clinical Immunology & Rheumatology, Academic Medical Center, Amsterdam, the Netherlands; 2 Department of Genome Analysis, Academic Medical Center, Amsterdam, the Netherlands; 3 Department of Experimental Immunology, Academic Medical Center, Amsterdam, the Netherlands; 4 Renal Transplant Unit, Academic Medical Center, Amsterdam, the Netherlands; 5 Bioinformatics Laboratory, Academic Medical Center, Amsterdam, the Netherlands; 6 Sanquin Research at CLB and Landsteiner Laboratory, Amsterdam, the Netherlands; Oregon Health Sciences University, United States of America

## Abstract

CD8^+^ T-cell responses against latent viruses can cover considerable portions of the CD8^+^ T-cell compartment for many decades, yet their initiation and maintenance remains poorly characterized in humans. A key question is whether the clonal repertoire that is raised during the initial antiviral response can be maintained over these long periods. To investigate this we combined next-generation sequencing of the T-cell receptor repertoire with tetramer-sorting to identify, quantify and longitudinally follow virus-specific clones within the CD8^+^ T-cell compartment. Using this approach we studied primary infections of human cytomegalovirus (hCMV) and Epstein Barr virus (EBV) in renal transplant recipients. For both viruses we found that nearly all virus-specific CD8^+^ T-cell clones that appeared during the early phase of infection were maintained at high frequencies during the 5-year follow-up and hardly any new anti-viral clones appeared. Both in transplant recipients and in healthy carriers the clones specific for these latent viruses were highly dominant within the CD8^+^ T-cell receptor Vβ repertoire. These findings suggest that the initial antiviral response in humans is maintained in a stable fashion without signs of contraction or changes of the clonal repertoire.

## Author Summary

Several viruses have found ways to evade the human immune system and cause latent infections. Examples include HIV and herpes-viruses. Most humans carry these herpes-viruses. The human immune system mounts continuous responses against these viruses to prevent them from causing disease. If this balance is disturbed, these viruses can cause extensive pathology. We do not know how the immune response against these viruses evolves over time. Understanding this response might help to understand why the immune system does not clear these viruses and might help in preventive and therapeutic strategies. Here we used a new technology that allowed us to track virus specific immune cells (CD8^+^ T cells) over time in a quantitative manner. When we used this technology to study the evolution of latent responses against herpes-viruses (from infection until 5 years later) we found that immune responses were very rigid and did not evolve over time. Collectively our data shows that – for these herpes-viruses – the initial immune response is maintained despite the fact that this does not result in clearance of the virus. Therefore, if a virus survives the initial response, it will not be cleared in the future. This is an important consideration in understanding latent infection and for vaccination-design.

## Introduction

The selection and maintenance of CD8^+^ T-cell clones is pivotal in antiviral immune responses. CD8^+^ T-cell responses can be divided in 3 different phases. After detection of a viral peptide, high-affinity CD8^+^ T-cells will clonally expand and acquire effector functions to clear the infected cells (early/expansion phase). After resolution of the infection, the pool of virus-specific clones will contract (contraction phase). The residual clones will be preserved for many years and are rapidly mobilized upon secondary infection with the same pathogen (memory phase).

Most of our current understanding of this process is based on animal models of infection that allow tight control over many factors such as viral titers and the exact time of infection [1], [2], [3]. Although these models are invaluable for our understanding of immune recognition and regulation, there are limitations in translating their findings to the human situation. A particular example is the CD8^+^ T-cell maintenance in persisting (or latent) viral infections (e.g. HIV, human cytomegalovirus (hCMV), and Epstein-Barr Virus (EBV)). Although animal models of persisting infections exist (e.g. LCMV, mCMV), these models can usually only be studied over a period of weeks to months, while in humans latent infections need to be controlled for many decades. Defective antiviral responses in immunocompromised patients can lead to severe disease and even death (e.g. AIDS). Thus, gaining more insight into CD8^+^ T-cell responses in persistent infections in humans will be instrumental in preventive and therapeutic strategies such as vaccine development.

The most used infections to study the CD8^+^ T-cell response in humans are hCMV and EBV. These viruses express stable immunodominant epitopes. Furthermore, it is possible to study the early phase of these infections (e.g. in organ transplant recipients or (rare) previously healthy, symptomatic patients). For hCMV it has been reported that the phenotype of virus-specific CD8^+^ T-cells changes over time from activated T cell (CD8^+^CD45R0^+^CD27^+^HLA-DR^+^) in the early phase to resting vigilant effector-type cell (CD8^+^CD45RA^+^CCR7^−^CD27^−^CD28^−^) during latency. In contrast, in healthy virus carriers EBV-specific CD8^+^ T cells mostly have an early/intermediate memory T-cell phenotype (CD8^+^CD45RA^−^,CCR7^−^,CD27dim,CD28^+^) [4], [5], [6]. For both viruses, it was assumed that the resulting populations are long-lived and represent a ‘steady situation’ in which viral latency is maintained by these populations [7], [8]. However, these assumptions were challenged by a recent publication that showed that the latent response in a murine CMV (mCMV) infection is not formed by long-lived CD8^+^ T cells but by short-lived effector cells that are continuously being replaced [9]. A proportion of these short-lived cells were shown to be recruited from the naive T-cell compartment. These data therefore suggest that cellular recruitment in a latent response may be dynamic, and might have features of an ongoing early antiviral response.

Thus, the question remained whether virus-specific CD8^+^ T-cell repertoires in responses to herpes viruses in humans are indeed stable or may show signs of flexibility over time. Two papers reported that EBV-specific CD8^+^ T-cell clones are stable over the first years of infection, while a third reported changing patterns over the first 2 years following infection [10], [11], [12]. In hCMV, one longitudinal study found initial skewing of the repertoire after the acute/early infection, but also demonstrated the persistence of a number of early clones over a period of 5 years [13]. One of the limitations in addressing the evolution of clonal responses is that until recently there was no technique available that could analyze the T-cell receptor repertoire at the level of individual clones in a numerical way [14], [15]. As the above studies necessarily relied on ‘cloning and sequencing’ to identify individual clones, no quantitative data exist on the evolution of the clonal response nor on the size of the individual clones within the CD8^+^ T-cell population. Here we combined tetramer sorting with quantitative next-generation sequencing (NGS) to identify and track the antiviral clones during the early and latent phases of infection. We found that in both hCMV- and EBV-infection the clonal repertoires in the early phase of infection are highly similar to those of the latent phase. In the viral latency phase, hCMV- and EBV-specific clones remain amongst the highest clones in the CD8^+^ T-cell population, illustrating the relentless and stable continuation of responses to these herpes viruses by the immune system.

## Results/Discussion

### Progression of viral infections and antiviral responses over a 5-year period

**Figure 1:**
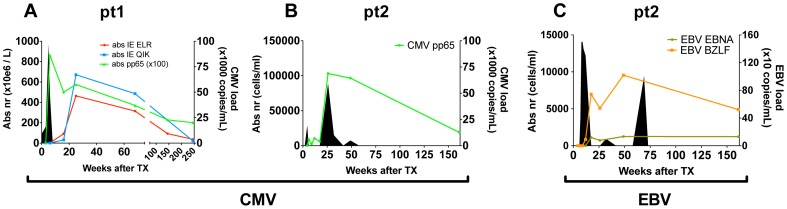
Overview of viral infections and CD8^+^ T cell responses following primary hCMV and EBV infection. (A/B) hCMV in pt1 and pt2. (C) EBV in pt2. Number of antiviral CD8^+^ T cells are shown per tetramer in solid lines (cell counts are depicted on the left axis). Viral loads are depicted by filled area's (viral copies are depicted on the right axis).

To study the progression of the antiviral T-cell responses we studied two hCMV- and EBV-seronegative patients who underwent primary hCMV-, respectively primary hCMV- and EBV-infection following transplantation with grafts from hCMV- and EBV-seropositive donors. In both patients viral loads and virus-specific CD8^+^ T-cell responses were measured over a 3–5 year period (Fig. 1). Details on patients and the infections are shown in table S1 in Text S1. In patient 1 (pt1) 3 different hCMV derived peptide-tetramer complexes were used to study the anti-viral responses against hCMV: HLA-B0801-ELRRKMMYM (CMV-IE-ELR) and HLA-B0801-QIKVRVDMV (CMV-IE-QIK), both derived from the Immediate Early (IE) protein I and HLA-A0101-YSEHPTFTSQY (CMV-pp65-YSE) derived from the tegument-protein pp65 (see table S2 in Text S1 for additional information on the tetramers). In pt2, we measured the response against hCMV using a HLA-B3501-IPSINVHHY tetramer (CMV-pp65-IPS). Additionally, in pt2 the EBV response was monitored with HLA-B3501-EPLPQGQLTAY (EBV-BZLF), derived from the lytic protein BZLF1, and HLA-B3501-HPVGEADYFEY (EBV-EBNA) derived from the non-lytic protein EBNA1. The phenotype of the virus-specific CD8^+^ T cells showed that the initial hCMV-response consisted primarily of memory-type CD8^+^ T cells (CD45RA^−^CD27^+^), which over time differentiated into effector-type CD8^+^ T cells (CD45RA^+^CD27^−^), while the EBV response consisted of early/intermediate CD8^+^ T cells (CD45RA^−^CD27^+^) during both early and latent phases (data not shown), which is in accordance with earlier reports [5], [13], [16].

#### Direct measurement of diversity and clonal size in the CD8^+^ population during the early phase of viral infection

**Figure 2:**
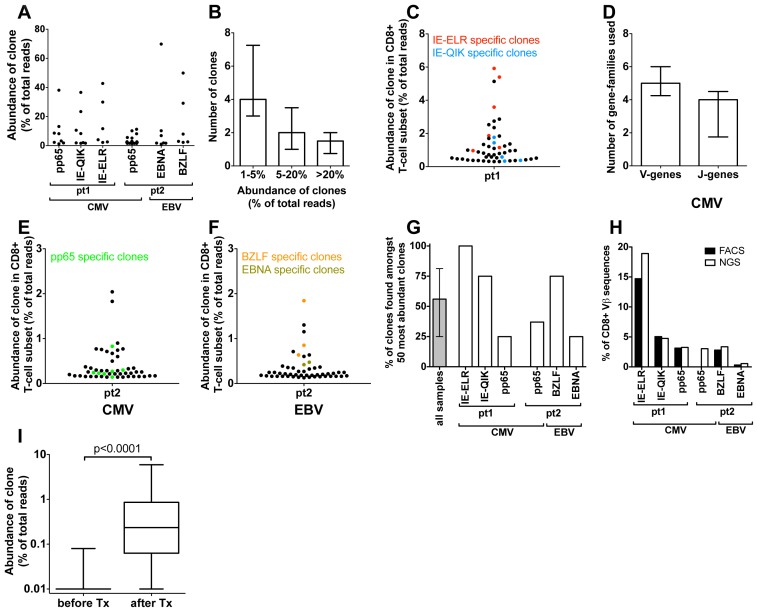
Quantification of clonal response in early response against hCMV and EBV. (A) Anti-viral clones (identified by tetramer sorting followed by NGS). Each dot represents a different clone. The degree of expansion was determined as the frequency of the individual clonal TCRß-sequence, expressed as percentage of the total number of TCRß-sequences analyzed. (B) Frequency distribution of anti-viral CD8^+^ T-cell clones (median and IQR). (C) Structural variation of antiviral clones, reflected in the number of different V- and J-genes used in each epitope response (median and IQR). (D–F). Using the unique TCRß sequence of each virus specific clones, they were identified within the total CD8 population during the early response (50 most abundant CD8^+^ T-cell clones are shown). Red = CMV-IE-ELR(-specific clones), blue = CMV-IE-QIK, green = CMV-pp65, tangerine = EBV-BZLF, asparagus = EBV-EBNA. (G) Percentage of tetramer-specific clones that could be detected amongst the 50 most abundant CD8 clones. Grey bar show median and IQR. (H) Comparison of percentage of CD8^+^ T cells that was tetramer specific as identified by FACS (black bars) to percentage of TCRß-reads that was attributable to tetramer-specific clones as determined by NGS (white bars). (I) Wilcoxon matched pairs test comparing the frequency of the 56 tetramer^+^ clones (all 6 tetramers combined) before and after infection (clones that were not detected were given a frequency of 1∶10,000 which was the lower detection limit).

We used a combination of FACS sorting and NGS of the TCR repertoire to quantitatively characterize the early antiviral response. We defined clones by their unique TCRß-sequence and included clones in the analyses if their TCRß-rearrangement made up >1% of the analyzed TCRß-sequences of the tetramer-sorted population. Using this approach we found a mean of 7.5 specific clones per tetramer (IQR = 6–11.5) (Fig. 2A). In 5 out of 6 tetramer responses there were 1 or 2 dominant clones that individually made up >20% of the TCRß sequences (Fig. 2B). This was similar for both hCMV and EBV. The structural variance of the responding clones was addressed by determining the V- and J-gene segments that were used by each clone (Fig. 2C). A median of five different V-gene variants (range 2–6) and 4 different J-genes variants (range 1–6) were observed per tetramer-sorted population. The variation in the responses against the BZLF-epitope (EBV) was much lower than in the other sorted populations (2 V-genes and 1 J-gene). These findings confirm earlier β-diversity estimates made by extensive (conventional) cloning and sequencing in hCMV (pp65 epitope) and EBV (BMLF epitope) in a quantitative way [10], [13]. Despite the observed diversity in the cited and the present studies, the findings clearly indicate that in most tetramer-sorted populations only 1 or 2 clones are dominating the responses.

Next we leveraged the quantitative deep-sequencing capacity of the NGS-protocol to identify the tetramer-specific clones amidst the total CD8^+^ T cell population, thereby determining their true degree of expression and rank with the full T-cell repertoire. To this end we sorted total CD3^+^CD8^+^ T cells from the same samples and sequenced their TCR repertoire. For each CD8^+^ T cell sorted sample >10,000 reads were obtained after sequencing and bioinformatics analysis. Using the TCRß sequence of each clone as identifier we could determine the frequency of each clone within the CD8^+^ T-cell population. Using this approach we could identify a median of 85% (IQR 77%–100%) of the virus-specific clones in the total CD8^+^ T-cell population (Fig. S1A–B in Text S1). The rank-order of the tetramer-sorted clones in the total CD8^+^ T-cell population corresponded well with their rank order in the tetramer sorted samples, confirming the quantitative read-out of this protocol (Fig. S1C–E in Text S1).

We found that many of the virus-specific clones could be detected amongst the most abundant clones in the CD8^+^ T-cell population. In pt 1, the 2 most abundant clones were directed against the IE-ELR peptide, while the 11^th^ and 13^th^ most frequent clones recognized the CMV-IE-QIK peptide (Fig. 2D). The most dominant hCMV pp65-specific clone was ranked 17^th^ (data not shown). These findings were confirmed in the second patient, where the 4^th^ clone in the CD8^+^ T-cell population was directed against the pp65-IPS tetramer, as well as 6 other clones in the 50 most abundant clones (Fig. 2E). For EBV a similar picture was found. During the early response, the 1^st^, 4^th^ and 6^th^ most frequent clones were directed against the BZLF peptide, while the 14^th^ most frequent clone was specific for the EBNA peptide (Fig. 2F). Overall a median of 56% (range 25–100%) of the tetramer responsive clones could be identified amongst the 50 most abundant clones (Fig. 2G). The clones from all 6 tetramer responses are shown in table S3 in Text S1. The EBV responses appeared more conserved than the CMV responses.

As our analysis uses mRNA copies as a proxy for the degree of expansion of the clones we wanted to confirm that this indeed gives an accurate representation. To this end we calculated which percentage of the CD8^+^ T-cell population was specific for a given tetramer as determined by FACS. Subsequently we calculated which part of the TCRß-repertoire of the CD8^+^ T-cell population could be attributed to that tetramer by adding up the TCRß-sequences that belonged to the tetramer-specific clones. The FACS and NGS findings corresponded well with each other for all the tetramers measured (Fig. 2H). This indicates that the dominance of the tetramer specific clones at the TCRß repertoire level is an accurate representation of the clones in term of cell numbers. These findings are consistent with previous reports that TCR mRNA levels are stable [17].

To see whether the clones detected by tetramers during the viral infection had any precursor frequency we sequenced the TCR repertoires of the (total) CD8^+^ T-cell samples. We found that of the 56 clones that were tetramer^+^ at the early time point (all 6 tetramers combined) 47 (84%) clones could not be detected prior to transplantation, which means that for most of the tetramer^+^ clones the precursor frequency is extremely low (at least lower than 1∶10,000, which was the detection limit in this experiment). The clones that were identified, only just made the detection limit (median 3/10,000 reads (IQR 1.8–3.6 reads/10,000). From this experiment we concluded that the vast majority of the tetramer^+^ clones identified during the early response had a very low precursor frequency. To formally test whether the precursor frequency was indeed lower than the frequency of the clones after the infection we performed a Wilcoxon matched pairs test comparing the frequency of all 56 clones before and after infection (clones that were not detected were given a frequency of 1∶10,000 which was the lower detection limit). We found that the median frequency of the clones before infection was 0.01% (IQR 0.01%–0.01%) compared to 0.23% (0.86%–0.06%, p<0.001) at the early stage of infection (Fig. 2I).

Collectively, our data show that the NGS-approach enables identification of individual antiviral T-cell expansions in the CD8^+^ T-cell populations. Moreover, this approach showed that, during primary infection, the hCMV- and EBV- specific clones are amongst the most abundant clones in the CD8^+^ T-cell population.

#### The clonal response against the EBV and hCMV epitopes does not show contraction in the years following infection and maintains its diversity

**Figure 3:**
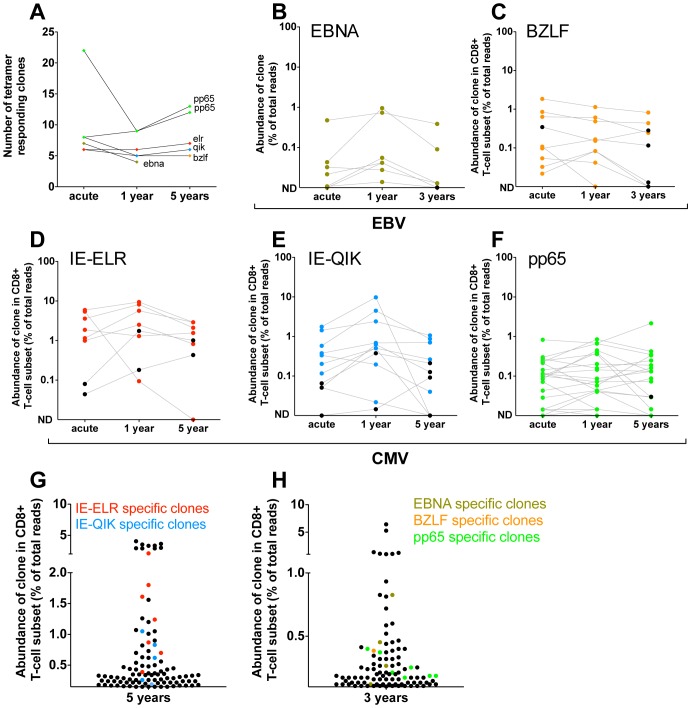
Quantification of clonal response in latent phase of response against hCMV and EBV. Clones were identified using tetramers loaded with peptides from immunodominant epitopes. All clones that had a frequency >1% of the TCR sequences were included. The frequency of each clone was determined within the CD8 population. (A) Number of tetramer specific clones during early phase of response (pt1: 6 wks for CMV-pp65, 16 wks CMV-IE-QIK and CMV-IE-ELR; pt2: 25 wks for CMV-pp65, 18 wks for EBV-BZLF and EBV-EBNA) and after 1 year, 3 year (EBV) en 5 year (hCMV) of follow-up. (B–F) Longitudinal follow-up of individual tetramer specific clones. Clones that were >1% in the tetramer sort at the early phase are colored Red = CMV-IE-ELR(-specific clones), blue = CMV-IE-QIK, green = CMV-pp65, tangerine = EBV-BZLF, asparagus = EBV-EBNA. Clones that were identified at later time point were retraced during the early phase (black). All these clones could be found at low frequency during the early phase. ND = not detected. (G–H) Rank of tetramer-specific clones among the 50 most abundant CD8 clones within the peripheral blood. Red = CMV-IE-ELR(-specific clones), blue = CMV-IE-QIK, green = CMV-pp65, tangerine = EBV-BZLF, asparagus = EBV-EBNA.

Next, we investigated whether the clonal composition of the antiviral repertoires would change in the years after the primary infection. First we identified the number of specific clones by tetramer sorting 1 and 3 years (pt2) after infection or 1 and 5 years (pt1) after primary infection (Fig. 3A). Most epitopes showed a constant number of responding clones when compared to the early phase. The median number of clones changed from 7.5 during the early response to 5.5 (IQR 5–9) after 1 year to 7 (IQR 5.5–12.5) after 3/5 year. Only the pp65-specific response in pt2 (that showed a polyclonal early response) showed lower diversity during the first year in this analysis. However, in this case 11 of the 22 clones found in the early phase were present in the tetramer after 1 year but their frequencies did not exceed the threshold of 1% of the reads. The same was seen for the 3-year follow-up sample of these epitope-specific clones. Therefore, for this tetramer-sorted population the threshold of 1% - that was used to exclude non-specific clones – may have been somewhat stringent.

Next, we focused on the longitudinal behavior of the individual clones (Fig. 3B–F). For this analysis we studied the frequencies of the virus-specific clonal TCR sequences in the CD8 T-cell population. Strikingly, after 1 year, a median of 95% (IQR 64–100%) of the tetramer-specific clones from the early response could be recovered within the CD8^+^ T cell population. After 3 (pt2) and 5 years of follow-up this was 77% (IQR 69–83%). Additionally, the hierarchy of the clones remained mostly unchanged, with the most dominant virus-specific clones retaining their positions in the hierarchy. As a logical consequence, only few ‘new’ clones were identified during the follow up period. In retrospect, all of these ‘new’ clones could be identified in the CD8^+^ T-cell population during the early phase, but their degree of presentation was low. This could be confirmed in the tetramer sorts of the early phase, where we retrieve most of these ‘new’ clones, but in a frequency <1% of the TCR-ß-reads. The ‘new’ clones represent a mean of 5.3% of the total responses after 1 year and 11.4% after 3–5 year follow-up. The fact that they are not abundantly present in the blood during the early phase raises the question whether they reside in a different compartment during the early phase (e.g. lymph nodes). Alternatively, these clones might undergo selective clonal expansion later on, but this would be counterintuitive as the viral epitopes do not change. Finally, it might simply be the result of fluctuations in the frequency that was also observed for some of the other clones. This effect is unlikely to be caused by the sequencing protocol as that has been extensively validated to detect quantitative differences [15].

The pp65 response in pt1 could not be followed over time in the CD8^+^ T-cell compartment as the frequency of the pp65 responsive clones dropped below the detection limit of 0.01% in the CD8^+^ T-cell population. However, the tetramer sort after 1 and 5 years of follow up showed that all clones from the early response could be detected after 1 and 5 years and that no new clones had appeared in the tetramer sorts, thereby confirming the data of the other 5 tetramers used (Fig. S2 in Text S1).

Finally, we addressed the rank order of the individual clones within the CD8^+^ T-cell population during the latent phase (Fig. 3H–G). In pt1 we found that after 5 years 9 of the 50 most abundant clones could be attributed to the CMV-epitopes studied. Collectively the CMV response made up 15% of the TCRs analyzed. In pt 2 we found 3 and 4 of the most abundant clones to be directed against EBV and hCMV peptides respectively. Collectively they formed 3.6% of the TCRs analyzed.

These findings show that the clonal repertoire does not change after the early phase, and virtually all clones are maintained at high frequencies. This suggests that immune responses against persistent viruses in the peripheral blood compartment are not characterized by a clear contraction phase. The latency phase seems a protracted version of the early phase, which is intuitive, as there is still antigen present. This view is compatible with earlier qualitative observations in EBV and hCMV that showed that many ‘early clones’ persist in the latent phase in humans [11], [12], [18]. Our data adds that it is virtually the complete repertoire – including the hierarchy – that is maintained. An important issue here is how the early phase should be defined. Consistently with Day et al and Annels *et al.* we observed constant repertoires from 6–16 weeks onwards. However, there are also findings that would support the idea that contraction takes place in an even earlier stage of infection. Day et al. analyzed clones in the very early stage of infection (2–4 weeks) and reported early skewing of hCMV-specific clones. Annels et al. also reported that many very early clones in EBV infection (day 12) could not be recovered after 3.5 years. Therefore we also tried to analyze very early timepoints. In our patients, there were very few tetramer^+^ CD8^+^ T cells (hundreds) at these timepoints. We did sequence these very early samples and found diverse repertoires that showed little overlap with samples taken with a week interval (data not shown). Given that the number of tetramer^+^ cells were so low (<0.1%) at these early time points, we could not exclude the possibility that non-hCMV-specific T cells may have contaminated these samples during FACS. Therefore, we cannot exclude the possibility that the very early response of circulating hCMV-specific CD8^+^ T cells is very broad and shrinks rapidly after the first weeks.

#### The clones against hCMV and EBV constitute the most abundant CD8 clones in blood of healthy persons

**Figure 4:**
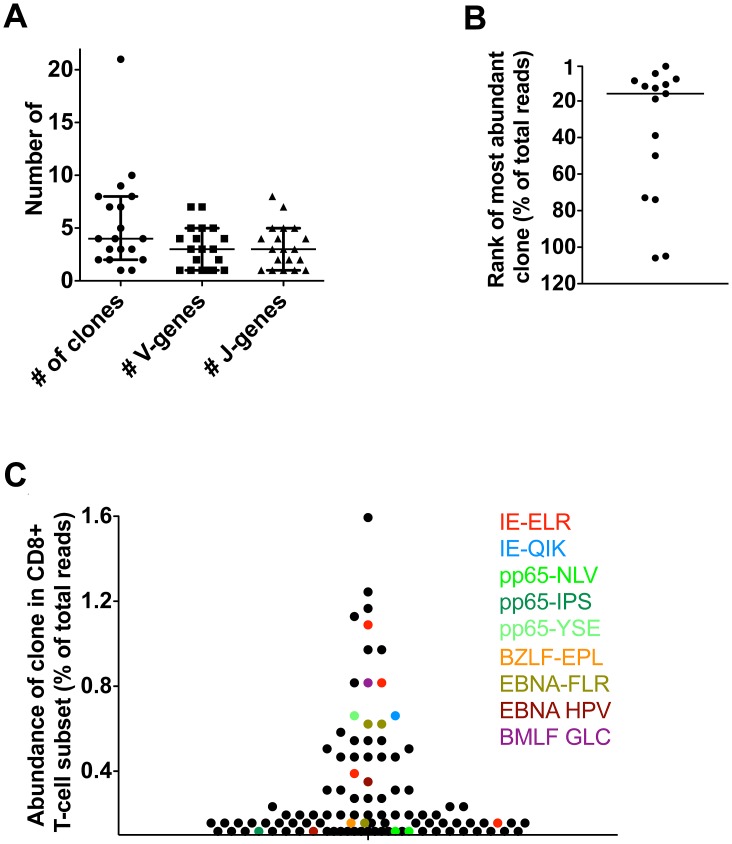
Latent phase anti-hCMV and EBV responses in healthy persons. Antiviral responses were measured in 5 healthy individuals using a panel of 14 tetramers. (A) Diversity of responses per tetramer; number of responding clones and number of V/J-genes used. (B) Rank of most abundant clone of each tetramer in the CD8 population. Median rank is 16^th^ (range 1^st^–106^th^). (C) Visualization of anti-hCMV and anti-EBV response in one HD using 9 tetramers.

Although the study of kidney transplant recipients offers the opportunity to study primary responses in a longitudinal fashion, it cannot be excluded that because of immunosuppressive medication these responses may differ from those in healthy people. Therefore, we next tested if similar patterns could be observed in healthy individuals during the latency phase of herpes virus infection. To this end, we included 5 healthy donors (HD) who were latently infected with hCMV and EBV. Responses were measured using 12 different tetramers. One of these persons responded to 9 different hCMV- and EBV-derived tetramers in our panel, while the others responded to 1–4 different tetramers. We found that a median of 4 clones was mounted against each epitope (range 1–21) (n = 19) (Fig. 4A). A median of 3 different V-genes (range 1–7) and 3 different J-genes (range 1–8) were found per tetramer response (Fig. 4A). Some tetramer-specific populations showed highly related CDR3s while others had a more diverse make-up (data not shown). Of interest, we found that the clonal repertoires against peptides derived from hCMV-IE were significantly less diverse than repertoires against hCMV-pp65 derived peptides, which could possibly be related to their different route of presentation [19] (Fig. S3 in Text S1). Therefore, the diversity of the response during the latent infection seems comparable with the ones observed in the transplant patients. Moreover, the phenotypes of the tetramer^+^ subsets were similar in patients and controls. (Figs. S4, S5, S6, S7 in Text S1). Previous longitudinal studies in healthy individuals readily showed, using conventional sequencing, that antiviral clones remain present during a follow-up of up to 4 years, which is in agreement with our findings in the transplant recipients [13], [18].

Finally, we investigated whether the virus-specific clones were amongst the highest clones in the total CD8^+^ T-cell pool, as found in the transplant recipients. To this end, we determined the highest rank of the tetramer responding clones within the CD8^+^ T-cell population. The median rank of the most dominant clones was 16^th^ within the CD8^+^ T-cell population (range 1–106). The healthy donor who responded to the 9 different tetramer-peptide complexes was used to visualize the impact of hCMV and EBV on the total CD8^+^ T-cell repertoire (Fig. 4C). In this individual, we observed that 6 of the 30 most abundant clones were directed against hCMV, mainly towards the IE epitope. These included the 5^th^ and 8^th^ most abundant clones. Many hCMV-pp65-specific clones were observed lower in the hierarchy. EBV specific clones made up 4 of the top 30 clones. Generally they were slightly lower in the hierarchy, the highest being ranked 9^th^. These findings show that also in healthy individuals many of the highly abundant clones are directed against epitopes of hCMV and EBV confirming our findings in the transplant recipients. Very large expansions of individual clones within the total CD8 T-cell repertoire detected by conventional sequencing have been reported previously [20], [21], [22].

Collectively, these data show that our mapping strategy using NGS can be used to dissect the CD8^+^ T-cell repertoire into individual responses. We showed in two latent viral infections in humans that latent anti-viral repertoires that are mounted early in the expansion phase are highly stable from the moment that they can be detected in blood until 3 to 5 years later. Importantly, most clones retained high ranks within the CD8^+^ T-cell repertoire.

Although we analyzed 6 different responses, a limitation of the current study is the number of transplant recipients included. Although the transplant setting allows for a unique opportunity to study primary anti-viral responses in man because the moment of infection is known, primary hCMV and especially EBV infection after transplantation are relatively rare events. Yet, studies of these unique transplant patients have steadily increased our insight into the evolution of anti-viral responses in humans [6], [8], [10], [13].

Recently, studies using a mouse model of a latent infection (mCMV) showed that the mCMV response is very dynamic and is maintained by the continuous recruitment of short lived effector cells [9]. Moreover the mCMV response also appeared to include the recruitment of naive cells into the response, although this was not a necessity for a successful response [9], [23], [24]. It was not analyzed if these naive population derived clones altered the clonal composition of the mCMV-specific CD8^+^ T-cell pool. Our findings do not suggest that there is a significant recruitment of naive clones in humans into the response as hardly any new clones appear in the anti-viral repertoires. Rather our data support a model of robust homeostatic maintenance of anti-viral memory and effector clones. This is in line with the finding that CD45RA^+^CD27^−^ effector T cells, although seldom dividing, are not prone to die [25]. Additionally, recent expression array analysis showed a strong transcriptional fingerprint in hCMV-specific effector CD8^+^ T cells, without signs of exhaustion, suggesting that these cells are long-term and active contributors to the antiviral response [26]. Nevertheless, we should notice that the maximal time span we could analyze in this study was 5 year and that when one considers that hCMV and EBV responses persist for decades low recruitment from the naive pool may eventually affect clonal compositions.

## Materials and Methods

### Patients/healthy donors

Two patients (55 and 24 years old) were included that got primary hCMV infection (pt 1) or both hCMV and EBV infection (pt 2) following kidney transplantation. They received basic immunosuppressive drug therapy consisting of prednisolon, cyclosporin and mycophenolate mofetil. Pt 2 additionally received CD25 mAb induction therapy on days 0 and 4 after transplantation. Neither of the patients showed signs of allograft rejection during the follow-up period. Peripheral Blood Mononuclear Cells (PBMCs) were obtained at several time points during early infection and after 1 and 5 years of follow-up. In addition, PBMCs from 5 healthy donors (HD) were included. These controls were selected based on EBV or hCMV positivity. All PMBC samples were isolated using density centrifugation and cryopreserved until analysis. The study was performed according to the Declaration of Helsinki and approved by the medical ethics committees of the Academic Medical Center-University of Amsterdam and Sanquin Blood Research at CLB and Landsteiner Laboratory (Amsterdam). All patients gave written informed consent.

### CMV-PCR, EBV-PCR, anti-CMV IgG, and anti-EBV IgG

Quantitative polymerase chain reaction (PCR) for hCMV and EBV was performed in EDTA (ethylenediaminetetraacetic acid) whole blood samples, as described [27]. To determine CMV serostatus, anti-CMV immunoglobulin G (IgG) was measured in serum using the AxSYM microparticle enzyme immunoassay (Abbott Laboratories, Abbott Park, IL, USA). EBV serostatus was determined by qualitative measurement of specific immunoglobulin G (IgG) against the viral capsid antigen (VCA) and against nuclear antigen of EBV using respectively the anti-EBV VCA IgG enzyme-linked immunosorbent assay and the anti-EBV nuclear antigen of EBV IgG enzyme-linked immunosorbent assay (Biotest, Dreieich, Germany). All tests were performed according to the instructions of the manufacturers.

### Immunofluorescence staining, flow cytometry

For the isolation of CD3^+^CD8^+^ virus-specific cells, PBMC were stained with the appropriate APC-labeled tetramer (supplementary table S2 in Text S1 for all tetramers) (Sanquin Reagents, Amsterdam, The Netherlands) followed by staining with CD8-PerCP Cy5.5 (BD Biosciences, San Jose, CA, USA) and CD3 PE-Cy7 (BD), and subsequently sorted on a FACsARIA (BD). Purity of the obtained cells was checked by flow cytometry analysis of at least 100 cells. If the purity was less than 95%, the sorted cells were subjected to a second sort. For the isolation of total CD3^+^CD8^+^ cells, PBMC were stained with CD8-PerCP Cy5.5 and CD3-PE-Cy7 and subsequently sorted on a FACsARIA. Purity of the obtained cells was checked by flow cytometry analysis of at least 1000 cells. If the purity was less than 95%, the sorted cells were subjected to a second sort.

### RNA/cDNA

RNA was isolated from the virus-specific sorted cells and sorted CD8^+^ T-cell subsets with the Nucleospin RNA xs kit (Machery Nagel, Düren, Germany). The RNA of virus-specific CD8^+^ T cells was subsequently subjected to template switch-anchored reverse transcriptase–polymerase chain reaction (RT-PCR) by using the Smarter pico cDNA PCR synthesis kit and the Advantage 2 PCR kit (both: Clontech, Mountain View, CA, USA) according to manufacturers instructions. 250 ng of RNA of the sorted CD8^+^ T cell subsets was used as input for cDNA synthesis using the Superscipt III cDNA system (Invitrogen). Synthesis was performed with oligo-dT primers according to the manufacturer's protocol in a total volume of 20 uL.

### Linear amplification & Next Generation Sequencing

The linear amplification procedure has been described before [15], [28]. Briefly, as input for the linear amplification we used either 10 uL of DNA of the SMARTer pico treated samples or 20 uL of ‘regular’ cDNA (equivalent to 250 ng of total RNA). The (c)DNA was amplified using a set of 23 primers that cover all functional Variable gene segments of the TCRβ-chain [29]. Here we will use the HUGO-nomenclature according to ref. [30]. Primers were purchased from Biolegio (Nijmegen, NL). All V-primers contained the primerB sequence on the 5′ as described in the manufacturer's instructions for Amplicon sequencing using the Genome Sequencer FLX Titanium system (Roche Diagnostics Mannheim, Germany). In the first step of linear amplification, the cDNA was amplified in the presence of 4 pmol of each V-primer, 1 mM MgCl_2_, 0.1 mM dNTP's, 1× buffer B (Solis BioDyne, Tartu, Estonia), and 1U of Hotfire-Polymerase (Solis BioDyne) in a volume of 20 µL. The linear amplification was performed on a T3000 thermocycler (Biometra, Goettingen, G) (96°C for 15 min, 40× (96°C for 30 s, 60°C for 30 s, 72°C for 60 s), 72°C for 10 min). Amplification-products were purified using AMPure SPRI beads (Agencourt Bioscience, Beverly, MA) in a ratio of bead∶product of 0.9 according to the manufacturer's protocol. After purification the TCRs were amplified with a generic PCR using the generic extension on V-side and a Cβ specific primer to prevent PCR-bias (The PCR was performed on 10 uL of amplified product in the presence of 10 pmol of the primers, 1 mM MgCl_2_, 0.1 mM dNTP's, 1× buffer B, and 3 U of Hotfire-Polymerase in a total volume of 40 µL on a T3000 thermocycler (96°C for 15 min, 40× (96°C for 30 s, 60°C for 30 s, 72°C for 60 s), 72°C for 10 min). After the PCR, the products were purified using Ampure beads in bead∶product ratio of 1∶1. Products were prepared for sequencing according to the manufacturer's instruction for Amplicon sequencing on the Genome Sequences FLX titanium platform (Roche). Next-Generation Sequencing was performed on a Roche Sequencer FLX using the Titanium platform.

### Bioinformatics & data-analysis

The bioinformatics pipeline used to obtain the TCR-sequence was described in a previous paper and contains 4 modules: (1) Multiplex IDentifier-sorting, (2) Blast Like Alignment Tool (BLAT) Identification of gene segments, (3) CDR3 detection, (4) removal of artifacts [15].

## Supporting Information

Text S1 **Supplementary material for Klarenbeek/Remmerswaal-PLoS-Pathogens-2012.** This file contains supplementary Figures S1, S2, S3, S4, S5, S6, S7 and supplementary Tables S1, S2, S3.
(DOCX)

## References

[1] Wherry EJ, Ahmed R (2004) Memory CD8 T-cell differentiation during viral infection. J Virol 78: 5535–5545. 15140950 10.1128/JVI.78.11.5535-5545.2004 PMC415833

[2] Wherry EJ, Blattman JN, Murali-Krishna K, van der Most R, Ahmed R (2003) Viral persistence alters CD8 T-cell immunodominance and tissue distribution and results in distinct stages of functional impairment. J Virol 77: 4911–4927. 12663797 10.1128/JVI.77.8.4911-4927.2003 PMC152117

[3] Wong P, Pamer EG (2003) CD8 T cell responses to infectious pathogens. Annu Rev Immunol 21: 29–70. 12414723 10.1146/annurev.immunol.21.120601.141114

[4] van Leeuwen EM, ten Berge IJ, van Lier RA (2007) Induction and maintenance of CD8+ T cells specific for persistent viruses. Adv Exp Med Biol 590: 121–137. 17191382 10.1007/978-0-387-34814-8_9

[5] Roos MT, van Lier RA, Hamann D, Knol GJ, Verhoofstad I, et al (2000) Changes in the composition of circulating CD8+ T cell subsets during acute epstein-barr and human immunodeficiency virus infections in humans. J Infect Dis 182: 451–458. 10915075 10.1086/315737

[6] Callan MF (2003) The evolution of antigen-specific CD8+ T cell responses after natural primary infection of humans with Epstein-Barr virus. Viral Immunol 16: 3–16. 12725684 10.1089/088282403763635401

[7] Waller EC, Day E, Sissons JG, Wills MR (2008) Dynamics of T cell memory in human cytomegalovirus infection. Med Microbiol Immunol 197: 83–96. 18301918 10.1007/s00430-008-0082-5

[8] van Lier RA, ten Berge IJ, Gamadia LE (2003) Human CD8(+) T-cell differentiation in response to viruses. Nat Rev Immunol 3: 931–939. 14647475 10.1038/nri1254

[9] Snyder CM, Cho KS, Bonnett EL, van Dommelen S, Shellam GR, et al (2008) Memory inflation during chronic viral infection is maintained by continuous production of short-lived, functional T cells. Immunity 29: 650–659. 18957267 10.1016/j.immuni.2008.07.017 PMC2583440

[10] Annels NE, Callan MF, Tan L, Rickinson AB (2000) Changing patterns of dominant TCR usage with maturation of an EBV-specific cytotoxic T cell response. J Immunol 165: 4831–4841. 11046006 10.4049/jimmunol.165.9.4831

[11] Callan MF, Annels N, Steven N, Tan L, Wilson J, et al (1998) T cell selection during the evolution of CD8+ T cell memory in vivo. Eur J Immunol 28: 4382–4390. 9862375 10.1002/(SICI)1521-4141(199812)28:12<4382::AID-IMMU4382>3.0.CO;2-Z

[12] Silins SL, Cross SM, Elliott SL, Pye SJ, Burrows SR, et al (1996) Development of Epstein-Barr virus-specific memory T cell receptor clonotypes in acute infectious mononucleosis. J Exp Med 184: 1815–1824. 8920869 10.1084/jem.184.5.1815 PMC2192868

[13] Day EK, Carmichael AJ, ten Berge IJ, Waller EC, Sissons JG, et al (2007) Rapid CD8+ T cell repertoire focusing and selection of high-affinity clones into memory following primary infection with a persistent human virus: human cytomegalovirus. J Immunol 179: 3203–3213. 17709536 10.4049/jimmunol.179.5.3203

[14] Bonarius HP, Baas F, Remmerswaal EB, van Lier RA, ten Berge IJ, et al (2006) Monitoring the T-cell receptor repertoire at single-clone resolution. PLoS One 1: e55. 17183685 10.1371/journal.pone.0000055 PMC1762342

[15] Klarenbeek PL, Tak PP, van Schaik BD, Zwinderman AH, Jakobs ME, et al (2010) Human T-cell memory consists mainly of unexpanded clones. Immunol Lett 133: 42–48. 20621124 10.1016/j.imlet.2010.06.011

[16] Gamadia LE, Remmerswaal EB, Weel JF, Bemelman F, van Lier RA, et al (2003) Primary immune responses to human CMV: a critical role for IFN-gamma-producing CD4+ T cells in protection against CMV disease. Blood 101: 2686–2692. 12411292 10.1182/blood-2002-08-2502

[17] Schrum AG, Turka LA, Palmer E (2003) Surface T-cell antigen receptor expression and availability for long-term antigenic signaling. Immunol Rev 196: 7–24. 14617194 10.1046/j.1600-065x.2003.00083.x

[18] Iancu EM, Corthesy P, Baumgaertner P, Devevre E, Voelter V, et al (2009) Clonotype selection and composition of human CD8 T cells specific for persistent herpes viruses varies with differentiation but is stable over time. J Immunol 183: 319–331. 19542443 10.4049/jimmunol.0803647

[19] Gilbert MJ, Riddell SR, Plachter B, Greenberg PD (1996) Cytomegalovirus selectively blocks antigen processing and presentation of its immediate-early gene product. Nature 383: 720–722. 8878482 10.1038/383720a0

[20] Weekes MP, Wills MR, Mynard K, Carmichael AJ, Sissons JG (1999) The memory cytotoxic T-lymphocyte (CTL) response to human cytomegalovirus infection contains individual peptide-specific CTL clones that have undergone extensive expansion in vivo. J Virol 73: 2099–2108. 9971792 10.1128/jvi.73.3.2099-2108.1999 PMC104454

[21] Weekes MP, Carmichael AJ, Wills MR, Mynard K, Sissons JG (1999) Human CD28−CD8+ T cells contain greatly expanded functional virus-specific memory CTL clones. J Immunol 162: 7569–7577. 10358214

[22] Trautmann L, Rimbert M, Echasserieau K, Saulquin X, Neveu B, et al (2005) Selection of T cell clones expressing high-affinity public TCRs within Human cytomegalovirus-specific CD8 T cell responses. J Immunol 175: 6123–6132. 16237109 10.4049/jimmunol.175.9.6123

[23] Torti N, Walton SM, Brocker T, Rulicke T, Oxenius A (2011) Non-hematopoietic cells in lymph nodes drive memory CD8 T cell inflation during murine cytomegalovirus infection. PLoS Pathog 7: e1002313. 22046127 10.1371/journal.ppat.1002313 PMC3203160

[24] Loewendorf AI, Arens R, Purton JF, Surh CD, Benedict CA (2011) Dissecting the requirements for maintenance of the CMV-specific memory T-cell pool. Viral Immunol 24: 351–355. 21721929 10.1089/vim.2010.0140 PMC3154399

[25] Maini MK, Gudgeon N, Wedderburn LR, Rickinson AB, Beverley PC (2000) Clonal expansions in acute EBV infection are detectable in the CD8 and not the CD4 subset and persist with a variable CD45 phenotype. J Immunol 165: 5729–5737. 11067931 10.4049/jimmunol.165.10.5729

[26] Hertoghs KM, Moerland PD, van Stijn A, Remmerswaal EB, Yong SL, et al (2010) Molecular profiling of cytomegalovirus-induced human CD8+ T cell differentiation. J Clin Invest 120: 4077–4090. 20921622 10.1172/JCI42758 PMC2964975

[27] Boom R, Sol C, Weel J, Gerrits Y, de Boer M, et al (1999) A highly sensitive assay for detection and quantitation of human cytomegalovirus DNA in serum and plasma by PCR and electrochemiluminescence. J Clin Microbiol 37: 1489–1497. 10203511 10.1128/jcm.37.5.1489-1497.1999 PMC84811

[28] van Gisbergen KP, Klarenbeek PL, Kragten NA, Unger PP, Nieuwenhuis MB, et al (2011) The Costimulatory Molecule CD27 Maintains Clonally Diverse CD8(+) T Cell Responses of Low Antigen Affinity to Protect against Viral Variants. Immunity 35: 97–108. 21763160 10.1016/j.immuni.2011.04.020

[29] van Dongen JJ, Langerak AW, Bruggemann M, Evans PA, Hummel M, et al (2003) Design and standardization of PCR primers and protocols for detection of clonal immunoglobulin and T-cell receptor gene recombinations in suspect lymphoproliferations: report of the BIOMED-2 Concerted Action BMH4-CT98-3936. Leukemia 17: 2257–2317. 14671650 10.1038/sj.leu.2403202

[30] Folch G, Lefranc MP (2000) The human T cell receptor beta variable (TRBV) genes. Exp Clin Immunogenet 17: 42–54. 10686482 10.1159/000019123

