# Supplementary material for: Deep Sequencing of Antiviral T-Cell Responses to HCMV and EBV in Humans Reveals a Stable Repertoire That Is Maintained for Many Years
Source: PLoS Pathog. 2012 Sep 27;8(9):e1002889. doi: 10.1371/journal.ppat.1002889 (PMC3460621; doi:10.1371/journal.ppat.1002889)
Supplement: Text S1 — Supplementary material for Klarenbeek/Remmerswaal-PLoS-Pathogens-2012. This file contains supplementary Figures S1, S2, S3, S4, S5, S6, S7 and supplementary Tables S1, S2, S3. (DOCX) [file ppat.1002889.s001.docx]

**Supplementary online material:**

PL Klarenbeek ^1,2,3^*, EBM Remmerswaal ^3,4^*, IJM ten Berge ^3,4^, ME Doorenspleet^1,2,3^, BDC van Schaik^5^, REE Esveldt^1,2,3^, SD Koch^3^, A ten Brinke^6^, AHC van Kampen^5^, FJ Bemelman^4^, PP Tak^1^, F Baas^2^, N de Vries^1^, RAW van Lier^3,6^.

^1^Depts. of Clinical Immunology & Rheumatology, ^2^Genome Analysis, ^3^Experimental Immunology; ^4^Renal Transplant Unit; ^5^Bioinformatics Laboratory, Academic Medical Center, Amsterdam, the Netherlands; ^6^Sanquin Research at CLB and Landsteiner Laboratory, Amsterdam, the Netherlands.

* contributed equally

**Content:**

Supplementary table S1, S2, S3

Supplementary figures S1, S2, S3, S4, S5, S6, S7

**Table 1: Patient characteristics & details on primary infections**

|  | **Patient 1** | **Patient 2** |
| --- | --- | --- |
| **Primary viral infection(s)** | CMV | CMV & EBV |
| **Age at moment of transplantion** | 24 | 55 |
| **Indication for kidney transplant** | autosomal recessive polycystic kidney disease | Morbus Alport |
| **Medication** | basiliximab (induction), Prednisolon, Mycophenolate Mofetil, Cyclosporine A, Myfortic | Prednisolon, Mycophenolate Mofetil, Cyclosporine A |
| **HLA-typing** | A01/11  B08/45 | A03  B35 |
| **Peak of early response (weeks after transplantation)** | CMV-pp65-YSE: 6  CMV-IE-QIK: 25  CMV-IE-ELR: 25 | CMV-pp65-IPS: 26  EBV-EBNA: 18  EBV-BZLF: 18 |
| **Sampling of acute response (weeks after transplantation)** | CMV-pp65: 6  CMV-IE-QIK: 16  CMV-IE-ELR: 16 | CMV-pp65: 25  EBV-EBNA: 18  EBV-BZLF: 18 |

**Table S2: Tetramers used in this study:**

| **Name** | **Peptide** | **Virus** | **Protein** | **HLA** |
| --- | --- | --- | --- | --- |
| **CMV IE ELR** | ELRRKMMYM | CMV | IE 1 | B*0801 |
| **CMV IE QIK** | QIKVRVDMV | CMV | IE 1 | B*0801 |
| **CMV pp65 YSE** | YSEHPTFTSQY | CMV | PP65 | A*0101 |
| **CMV pp65 NLV** | NLVPMVATV | CMV | PP65 | A*0201 |
| **CMV pp65 IPS** | IPSINVHHY | CMV | PP65 | B*3501 |
| **EBV BMLF GLC** | GLCTLVAML | EBV | BMLF-1 | A*0201 |
| **EBV BZLF EPL** | EPLPQGQLTAY | EBV | BZLF-1 | B*3501 |
| **EBV EBNA HPV** | HPVGEADYFEY | EBV | EBNA-1 | B*3501 |
| **EBV EBNA FLR** | FLRGRAYGL | EBV | EBNA-3A | B*0801 |
| **EBV BZLF RAK** | RAKFKQLL | EBV | BZLF-1 | B*0801 |

**Figure S1: Validation of tetramer-sequencing approach:** (a) Percentage of tetramer identified clones that were recovered in the CD8 population (these include both the samples at the acute infection and the samples after 1,3 and 5 year follow-up) (n=17). A median of 85% of the clones could be detected in the CD8 population. The clones from 2 samples (the 1 and 5 year follow-up samples of the CMV pp65 tetramer in pt1) were not detected indicating that the complete response had dropped below the detection limit of 0.01% of the CD8 population. (b) To ensure that all major antiviral clones were identified we focused on the abundance (degree of expansion) of the clones that were not recovered. Each dot represents a clone. We could confirm that the clones that we not picked up were amongst the least abundant clones of the tetramer sorts indicating that the frequency of these clones in the CD8 population had dropped below the detection limit. (c-e) Examples of hierarchy of clones within tetramer sort and within CD8 population: In each column a dot represent a tetramer specific clone. Connected dot represent the same clone, measured in both the tetremar sorted and the CD8 population. The hierarchy of the clones is virtually identical in the tetramer sort and the CD8 population, validating the frequencies in the CD8 populations correspond to the tetramer sorts and vice versa.

**Figure S2: Longitudinal follow-up of pp65 specific clones:** Although the frequency of the pp65 clones was too low to be visualized in the CD8 population, we could perform the follow-up in the tetramer-sorted population. Each red dot represents 1 clone. Identical clones at the different time points are connected by a gray line. We selected all clones that consisted of >1% of the TCR sequences at each time point and determined the frequency at the other time points. There was no loss or gain of new clones during the 5 years of follow-up. The most dominant clone from the acute response stayed dominant over the 5-year period.

**Table S3: Clones detected at early timepoint in kidney transplant patients. Tetramer, V-gene, J-gene and frequencies in tetramer+ subset and matched CD8 samples are shown**

| pt | tetramer | V-gene | CDR3 | J-gene | Frequency in tetramer+ subset (% of total) | Frequency in CD8 (% of total) |
| --- | --- | --- | --- | --- | --- | --- |
| pt 1 | CMV-pp65 | 27 | CASSTGDHSNQPQHFG | 3.2 | 38.11 | 0.90 |
|  |  | 5.6 | CASSLGVDFNYGYTFG | 1.2 | 13.19 | 0.36 |
|  |  | 29.1 | CSVTGTSYEQYFG | 2.7 | 8.69 | 0.12 |
|  |  | 15 | CATSSPGLASDEQFFG | 2.1 | 8.24 | 1.08 |
|  |  | 7.2 | CASSSPLLSSDTQYFG | 2.3 | 3.29 | 0.36 |
|  |  | 27 | CASSLSGGLWSYEQYFG | 2.7 | 2.28 | not detected |
|  |  | 29.1 | CSVVGRYYEQYFG | 2.7 | 1.92 | 0.06 |
|  |  | 24.1 | CATSGPSASYEQYFG | 2.7 | 1.28 | 0.24 |
|  |  |  |  |  |  |  |
|  | CMV-ELR | 5.1 | CASSFNGPNTEAFFG | 1.1 | 44.38 | 5.40 |
|  |  | 10.3 | CAISEFGLAGSDEQFFG | 2.1 | 27.56 | 5.92 |
|  |  | 27 | CASSLFDGTGSTEAFFG | 1.1 | 11.93 | 3.59 |
|  |  | 27 | CASSQGGSYNEQFFG | 2.1 | 3.80 | 1.15 |
|  |  | 19 | CASSLTQGANTEAFFG | 1.1 | 2.48 | 1.87 |
|  |  | 11.2 | CASSLYEEVLLSYNEQFFG | 2.1 | 2.09 | 1.00 |
|  |  |  |  |  |  |  |
|  | CMV-QIK | 4.1 | CASSPTPGQRNGYTFG | 1.2 | 36.64 | 1.76 |
|  |  | 7.8 | CASSLKPGLSIDTQYFG | 2.3 | 23.62 | 1.45 |
|  |  | 24.1 | CATSVPSGRSYNEQFFG | 2.1 | 10.61 | 0.38 |
|  |  | 12.3 | CASSRAGAYNEQFFG | 2.1 | 8.29 | 0.58 |
|  |  | 2 | CASSGVEGITVNTEAFFG | 1.1 | 2.03 | 0.20 |
|  |  | 7.2 | CASSRLAGADTQYFG | 2.3 | 1.93 | 0.33 |
|  |  | 7.2 | CASSLKPGLSIDTQYFG | 2.3 | 1.73 | 0.01 |
|  |  | 7.8 | CASSRLAGADTQYFG | 2.3 | 1.65 | 0.05 |
|  |  |  |  |  |  |  |
| pt 2 | EBV-EBNA | 5.5 | CASNRESAYTFG | 1.2 | 69.70 | 0.47 |
|  |  | 14 | CASREKGYDEQFFG | 2.1 | 10.55 | 0.01 |
|  |  | 24.1 | CATSGRYGEQFFG | 2.1 | 6.98 | 0.04 |
|  |  | 4.1 | CASSQAGVEQFFG | 2.1 | 1.93 | 0.02 |
|  |  | 4.1 | CASSQAGVEQYFG | 2.7 | 1.77 | not detected |
|  |  | 4.1 | CASSPREMLYNEQFFG | 2.1 | 1.70 | not detected |
|  |  | 7.7 | CASSLKTGELVFG | 2.2 | 1.10 | not detected |
|  |  |  |  |  |  |  |
|  | EBV-BZLF | 10.3 | CAISTGDSNQPQHFG | 1.5 | 49.84 | 1.82 |
|  |  | 6.6 | CASSTGDHSNQPQHFG | 1.5 | 31.87 | 0.69 |
|  |  | 10.3 | CATGTGDSNQPQHFG | 1.5 | 7.99 | 0.87 |
|  |  | 6.6 | CAISTGDSNQPQHFG | 1.5 | 3.04 | 0.03 |
|  |  | 10.3 | CASSTGDHSNQPQHFG | 1.5 | 2.62 | not detected |
|  |  |  |  |  |  |  |
|  | CMV-pp65 | 6.2 | CASSHTGAEAFFG | 1.1 | 11.27 | 0.83 |
|  |  | 6.2 | CASSPTTAYEQYFG | 2.7 | 10.21 | 0.24 |
|  |  | 12.3 | CASSPALDAQYFG | 2.3 | 8.17 | 0.23 |
|  |  | 11.3 | CASSPDLGSTEAFFG | 1.1 | 5.61 | 0.30 |
|  |  | 11.3 | CASSLDLGSTEAFFG | 1.1 | 5.25 | 0.11 |
|  |  | 27 | CASSSGGVYEQYFG | 2.7 | 2.93 | 0.11 |
|  |  | 6.2 | CASSLTGSEAFFG | 1.1 | 2.84 | 0.10 |
|  |  | 12.3 | CASSLALDEQFFG | 2.1 | 2.70 | 0.27 |
|  |  | 6.2 | CASSLTGSEAFFG | 1.1 | 2.66 | 0.04 |
|  |  | 29.1 | CSVMRTDFDQYFG | 2.7 | 2.41 | not detected |
|  |  | 6.2 | CASSYTGSEAFFG | 1.1 | 2.38 | 0.01 |
|  |  | 29.1 | CSVMRTDFQYFG | 2.7 | 2.30 | 1.43 |
|  |  | 12.3 | CASSLALDEQFFG | 2.1 | 2.28 | 0.14 |
|  |  | 6.2 | CASSGTGTEAFFG | 1.1 | 2.16 | 0.21 |
|  |  | 29.1 | CSVMRGEHDEQYFG | 2.7 | 1.97 | 0.09 |
|  |  | 6.2 | CASSYSTAGELFFG | 2.2 | 1.84 | 0.11 |
|  |  | 6.2 | CASSPGTEQFFG | 2.1 | 1.76 | not detected |
|  |  | 6.2 | CASSLTGTEAFFG | 1.1 | 1.65 | 0.03 |
|  |  | 6.2 | CCCTGACAGGC | 2.2 | 1.62 | not detected |
|  |  | 6.2 | CASSKTGTEAFFG | 1.1 | 1.62 | 0.10 |
|  |  | 6.2 | CASSYSTGQLQHFG | 1.5 | 1.57 | 0.07 |
|  |  | 6.2 | CASSPTGGELFFG | 2.2 | 1.49 | 0.01 |

**A**

**B**

**Figure S3: Diversity of clones per protein.**

(A) frequency of all clones detected in the tetramer sorts of 5 patients pre transplantation (692, 670, 516, 613 and 532) and 5 HD (23, 24, 25, 26 and 61) (% of TCRß-sequences after sequencing within the tetramer sorted population). Each color represents a different clone within the analysed population (B) The Simson index of diversity was performed as described earlier (Venturi et al., *J Immunol Methods*, 2007, ). A high diversity index corresponds to a high diversity. Surprisingly CMV-pp65 specific CD8 T cells had a higher diversity than CMV-IE specific CD8 T cells. Perhaps this is caused by the fact that IE can only be cross-presented since antigen processing and presentation of IE is blocked by CMV-pp65 (Gilbert et al., *Nature*, 1996, p270-272). For EBV-specific CD8 T cells no significant difference could be found, but it can not be excluded that when more EBV-specific CD8 T cell population are analyzed, the diversity between CD8 T cells directed against lytic and non-lytic EBV epitopes might be different. (bars are SEM, statistic test=Student T-test).


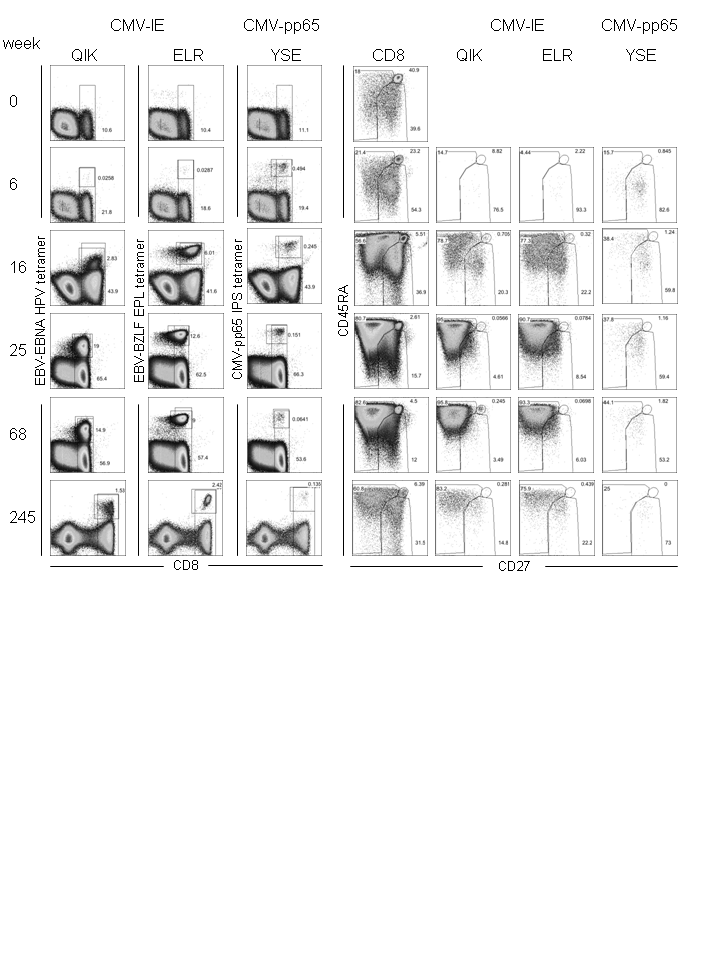


**Figure S4:** **Phenotyping of total CD8^+^ T cells and CMV-specific CD8^+^ T cells in pt 1**

Left panel: CD8 versus tetramer plots of all tetramers. Right panel: CD27 APC-Alexa Fluor 780 (eBioscience) and CD45RA PE-Cy7 (BD Biosciences) staining of total CD8^+^ T cells and CMV-IE QIK-, CMV-IE ELR- and CMV-pp65 YSE-specific CD8^+^ T cells. In the right panel subpanel are omitted if no tetramer+ cells were detected above background level.


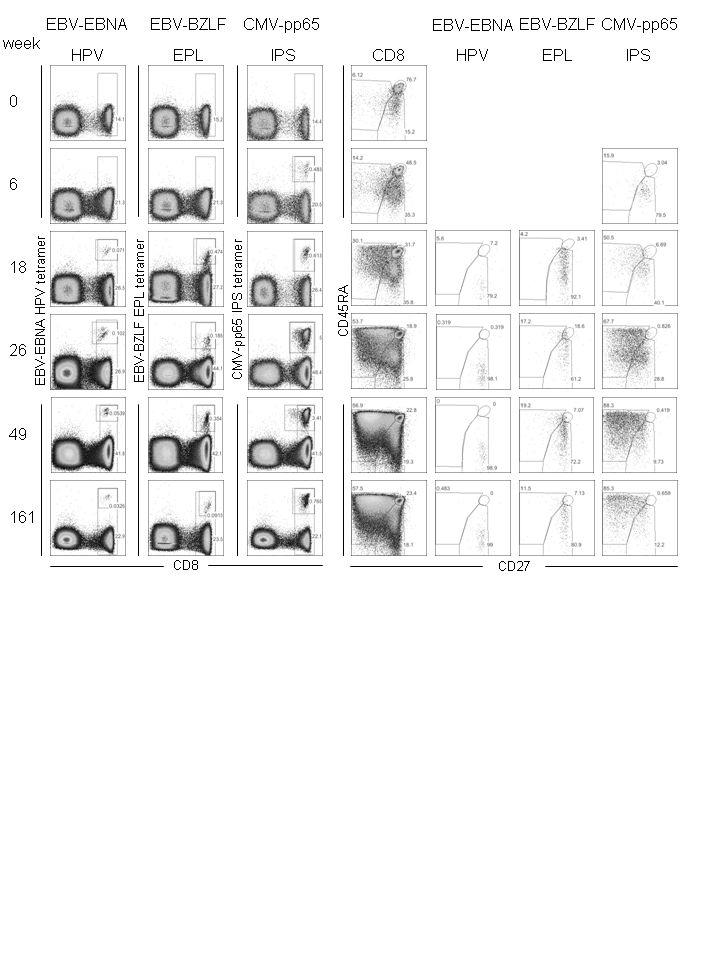


**Figure S5: Phenotyping of total CD8^+^ T cells and CMV and EBV-specific CD8^+^ T cells in pt 2**

Left panel: CD8 versus tetramer plots of all tetramers. Right panel: CD27 APC-Alexa Fluor 780 (eBioscience) and CD45RA PE-Cy7 (BD Biosciences) staining of total CD8^+^ T cells and EBV-EBNA HPV-, EBV-BZLF EPL- and CMV-pp65 IPS-specific CD8^+^ T cells. In the right panel subpanel are omitted if no tetramer+ cells were detected above background level.


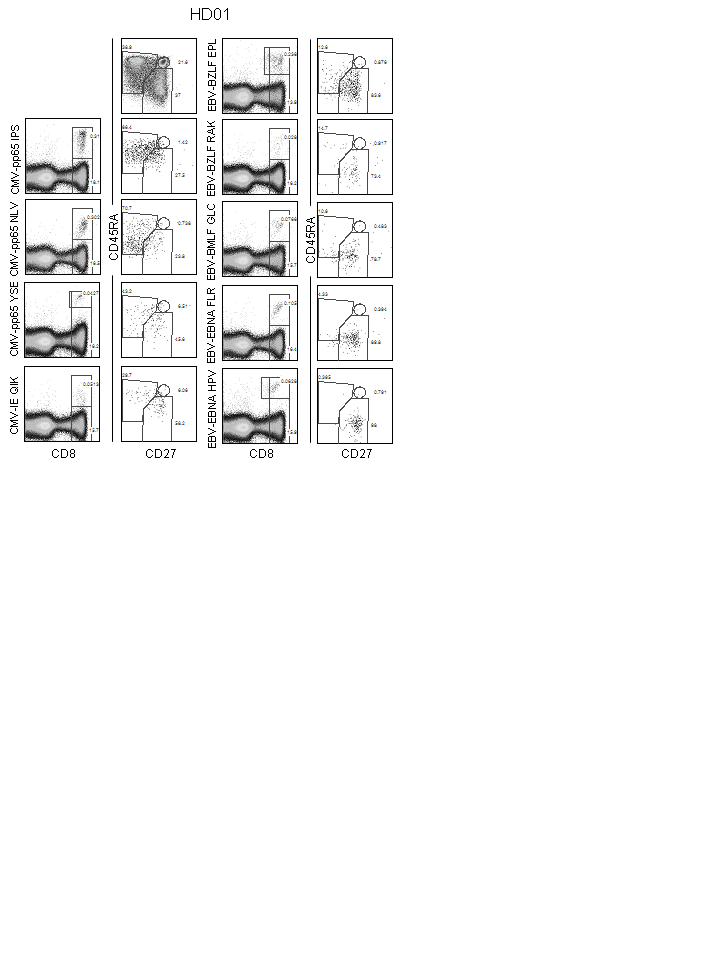


**Figure S6: Phenotyping of total CD8^+^ T cells and CMV, EBV and FLU-specific CD8^+^ T cells in HD01**

Left panels: CD8 versus tetramer plots of all tetramers. Top panel is gated on total CD8+ T cells. Right panels: CD27 APC-Alexa Fluor 780 (eBioscience) and CD45RA PE-Cy7 (BD Biosciences) staining of total CD8^+^ T cells and virus-specific CD8^+^ T cells.


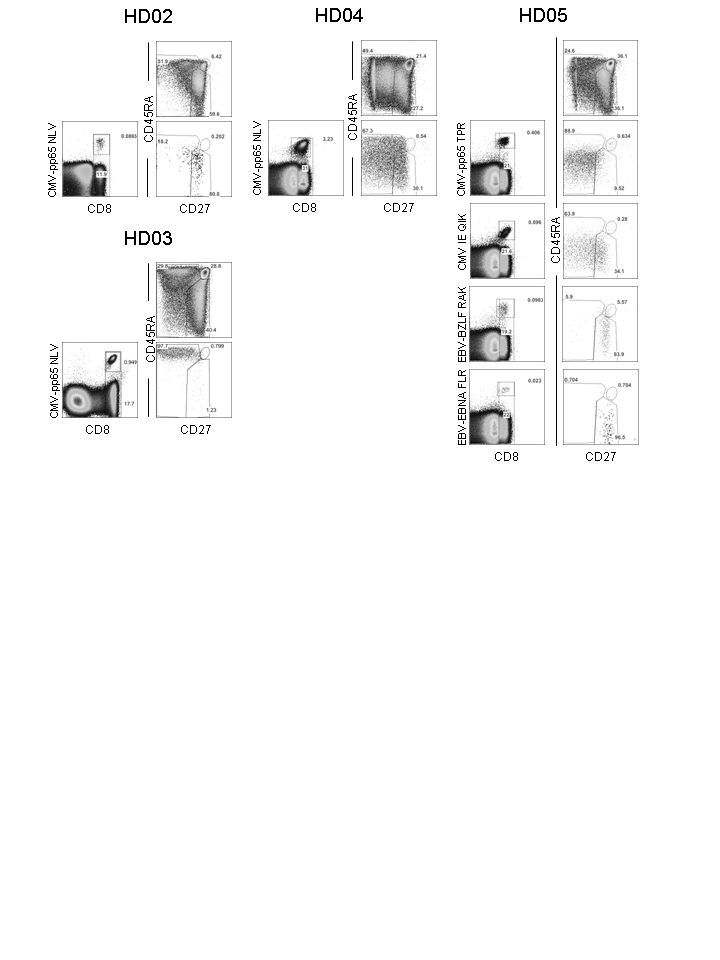


**Figure S7: Phenotyping of total CD8^+^ T cells and CMV, EBV and FLU-specific CD8^+^ T cells in HD02-05**

Left panels: CD8 versus tetramer plots of all tetramers. Right panels: CD27 APC-Alexa Fluor 780 (eBioscience) and CD45RA PE-Cy7 (BD Biosciences) staining of total CD8^+^ T cells and virus-specific CD8^+^ T cells.
